# Public Perception and Hand Hygiene Behavior During COVID-19 Pandemic in Indonesia

**DOI:** 10.3389/fpubh.2021.621800

**Published:** 2021-05-13

**Authors:** Ni Made Utami Dwipayanti, Dinar Saurmauli Lubis, Ngakan Putu Anom Harjana

**Affiliations:** ^1^Department of Public Health and Preventive Medicine, Faculty of Medicine, Udayana University, Denpasar, Indonesia; ^2^Faculty of Medicine, Center for Public Health Innovation, Udayana University, Denpasar, Indonesia; ^3^Institute for Population and Social Research, Mahidol University, Nakorn Pathom, Thailand

**Keywords:** hand hygiene, COVID-19, psychosocial factors, behavior, online survey

## Abstract

Hand hygiene practices are important not only during the corona virus disease 2019 (COVID-19) pandemic, but also critical to prevent the possible spread of other infectious diseases. This study aims to examine the current hand hygiene behaviors during the COVID-19 pandemic, post pandemic behavior intentions, and the relationship between behavior, psychosocial and contextual factors. A cross-sectional online survey was conducted from 28 May to 12 June 2020, with 896 valid responses obtained from Indonesian citizens over 18 years old. The survey questions included demographic characteristics, individual practices, risk perceptions, attitude, norm factors and ability factors related to hand hygiene during the COVID-19 pandemic. Descriptive analysis, chi square and multiple logistic regression tests were used to analyse the data. The results showed that 82.32% of female respondents and 73.37% male respondents reported handwashing practice 8 times or more per day during COVID-19 pandemic. Participants who perceived themselves at higher risk of contracting SARS-CoV-2 (OR 7.08, 2.26–22.17), had less negative perception toward the practice (OR 1.93, 1.32–2.82), perceived handwashing as an effective preventive measure (OR 1.77, 1.23–2.54), were female (OR 1.71, 1.21–2.41), perceived a more supportive norm (OR 1.68, 1.15–2.44) and noticed more barriers in access to handwashing facilities (OR 1.57, 1.05–2.36) were more likely to engage in hand hygiene practice more frequently during the pandemic. In conclusion, the majority of respondents did increase their frequency of hand hygiene practices during COVID-19 pandemic. In line with previous studies in other pandemic contexts, sex, perceived susceptibility and effectiveness are important predictors of hand hygiene practices, which are similar to findings from previous studies in other pandemic contexts. Addressing social norm related to the perceived hand hygiene practices of friends and important people is a potential health promotion strategy by creating hand hygiene norms in the community.

## Introduction

Corona virus disease 2019 (COVID-19) is a disease caused by severe acute respiratory syndrome coronavirus 2 (SARS CoV-2), a pathogen similar to SARS coronavirus that also causes respiratory disease (1). People with COVID-19 can suffer from mild infection to very severe disease. The SARS CoV-2 is carried in the nasopharynx, therefore spreading mainly through saliva droplets or nasal discharge when an infected person coughs or sneezes (2). The first cases of COVID-19 in Indonesia were announced by President Jokowi on March 2, 2020 in Jakarta. By 19th October, the Indonesian COVID-19 task Force reported 361,867 confirmed cases, 125,111 COVID-19 related deaths and 285,324 people recovered from COVID-19 (3). The Government of Indonesia has subsequently recommended a strategy to prevent transmission by performing the “three M's,” which stand for: *memakai masker* (using a mask), *menjaga jarak* (maintaining physical distance of 1 to 1.5 m) and *mencuci tangan pakai sabun* (handwashing with soap).

Handwashing with soap (HWWS) has actually been suggested by the World Health Organization as the most effective and low-cost strategy to prevent SARS CoV-2 transmission (4). A recent study reported that hand hygiene together with other protective measures such as wearing mask and avoiding the crowd have also contributed to the decrease in other respiratory infections during COVID-19 pandemic (5). Moreover, a substantial amount of peer-reviewed literature has shown the benefits of hand hygiene to prevent many infectious diseases including gastrointestinal illnesses (6–10); trachoma and soil helminth infection (11, 12) as well as respiratory infection (6). Thus, hand hygiene practices are not only important during a pandemic, but also critical to prevent the spread of other diseases.

In order to better understand factors that promote hand hygiene practices as a public health measure, it is beneficial to examine the community's behaviors through behavioral change theories such as the *Health Belief Model* and *Theory of Planned behavior* (TPB) (13, 14). The *TPB* highlights the importance of someone having a strong desire to change (intention) prior to achieving a behavior change. A desire to change is influenced by several groups of factors, namely attitudes toward a behavior, subjective norms and perceived behavior control (15). Mosler (16) developed the Risk, Attitude, Norm, Ability and Self-Regulating (RANAS) model based on these theories design a behavior change program in the area of water, sanitation, and hygiene (WASH). The RANAS model posits that, there are five groups of psychosocial factors which may influence WASH related behavior change, i.e., perceptions of risk, attitude factors, norm factors, ability factors and self-regulation factors (16, 17). These elements are modifiable by contextual factors such as, social, physical and personal factors (17). In a health behavior study, perceived risk was measured by three dimensions i.e., a likelihood of harm, susceptibility to illness and severity (18, 19). Attitude factors include the perception of benefits and the negative impact of the behavior (17). Health behaviors are also strongly influenced by social norms, which describe other's perceptions on behavior, thus creating social pressure to perform certain behavior (17, 20, 21). Ability and self-regulation factors represent an individual's confidence to perform and will to maintain the behavior (17).

Previous studies on preventive health behavior during the SARS-CoV epidemic and during the peak of the H1N1 epidemic have identified factors influencing the adoption of behavior which include: perceived likelihood for infection (22–24), perceived severity if contrating the disease and perceived effectiveness of the preventive behavior (23–25), and perceived ability to perform the behavior (24). Other contextual factors such as sex and age were also found to modify the preventive behavior (23, 25). However, many of these studies had limited attention to the negative perception toward the behavior and factors related to social norms that influence hand hygiene behavior among the general population. Other water, sanitation and hygiene (WASH) related studies have shown that this social norm is an important factor that can trigger and sustain behavior change (20, 26) and thus this factor should also be incorporated into post-pandemic WASH planning.

Although prevention measures introduced during the COVID-19 pandemic increased compliance with hand hygiene practices, it is important to understand how to sustain this practice in the post-pandemic period. Currently, the extent to which the COVID-19 pandemic has changed hand hygiene practices amongst the general population in Indonesia has not been systematically examined. Hence, this study is designed to explore three questions: the current situation and changes of hygiene behavior during the pandemic; behavior intention in the future of post-pandemic era; and the relationship between behavior and psychosocial factors (risk factor, attitude factors, norm factors and ability factors) as well as contextual factors. Understanding these factors is necessary to improve hand hygiene promotion or programming aiming for sustained behavior change for better prevention and management of possible disease outbreaks in the future.

## Methods

### Respondents and Procedures

A cross-sectional online survey was conducted from 28 May to 12 June 2020 as an exploratory investigation of handwashing practices during the COVID-19 pandemic in Indonesia using a convenience sampling technique. The respondents were Indonesian citizens currently living in Indonesia and over 18 years old, recruited through announcements posted in social media platforms such as WhatsApp groups and Facebook.

An incentive of IDR 250,000 (US$ 15.7) was given to 20 randomly selected respondents by using a lucky draw to attract more response to the survey. The respondents who were willing to participate accessed an online survey platform via a link provided in the announcement and completed the self-administered survey. There were 896 valid responses from a total 951 responses obtained from the survey. Ethical clearance for the study was obtained from Ethics Committee of Faculty of Medicine, Udayana University, Number 1170/UN14.2.2.VII.14/LT/2020.

### Instrument and Measurements

The questionnaire was developed based on the RANAS model on psychosocial factors relating to WASH behavior (16, 17) as well as previous studies related to hand hygiene behaviors and perceptions on health behavior (22–24). Subsequently, the questionnaire was piloted and revised based on trial feedback. Then, an online questionnaire was created using Google Form. All questions were set as required to be answered to prevent incomplete information and missing data. The five-part questionnaire included sections on demographic and information on settlement type; hand hygiene practices; psychosocial factors, including various perceptions toward hand hygiene; handwashing intention after the pandemic period; and sources of information related to health behavior to prevent COVID-19. A summary of the questions and the scale used for measurement is included in Supplementary Table 1.

Risk factors comprise of two variables: perceived susceptibility of being infected by SARS CoV-2 and perceived severity of if infected by SARS-CoV-2, where each was assessed with one question. Attitude factors consisted of two variables namely perceived effectiveness, which was measured with two questions on perceived effectiveness of hand hygiene preventing COVID-19 and other diseases, and negative attitude which was assessed with two questions on perceptions that handwashing is wasting water and wasting time. Perceived norm was assessed with three questions that included the perception of friends and other important people practicing more frequent hand hygiene, wearing a mask and maintaining social distance during COVID-19, perceptions of the need to be a good role model to others, and the perception that handwashing is part of religious norms. Ability factors consisted of perceived barriers which were assessed with questions on experience in accessing handwashing facilities outside their homes or in public places. To improve construct validity, questions used for each variable were tested with Pearson validity test and Cronbach's Alpha reliability test (Supplementary Table 2).

Respondents were also asked on intention to maintain hygiene behavior post COVID-19 and their perception about risk when the COVID-19 pandemic is over. At the end of the questionnaire, respondents were asked about the media that were frequently accessed for information regarding hygiene practices. The data was handled with care and confidentiality was ensured by only allowing research staff to access raw data.

### Data Handling and Analysis

Data analysis was conducted using Stata version 12.0 statistical software. Chi-squared tests were performed to assess the differences between variables related to handwashing practices and perceptions by sex and education level. Variables with *p* ≤ 0.05 in Chi-squared test was considered tone significantly different. For education variables, we classified secondary education or below as low education and tertiary education as high education. We conducted a comparative analysis to seek confounding and effect modification. Chi-square tests and multiple logistic regression models were used to assess a potential correlation of demographic variables and perceptions variables with hand hygiene frequencies, in which variables with *p* ≤ 0.05 are considered has a correlation. We re-classified categories of hand hygiene frequencies <8 times per day as low hygiene practice and frequencies of eight or more times per day as high hygiene practice. Variables of perceived effectiveness, negative attitude and perceived norm were re-categorized into two groups based on the median of the total score. This multiple logistic regression model was adjusted with socio-demographic characteristics such as sex, education and age. Variables with *p* ≤ 0.05 in multiple logistic regression were considered as independent predictors.

## Results

### Demographic Characteristic

**Figure 1:**
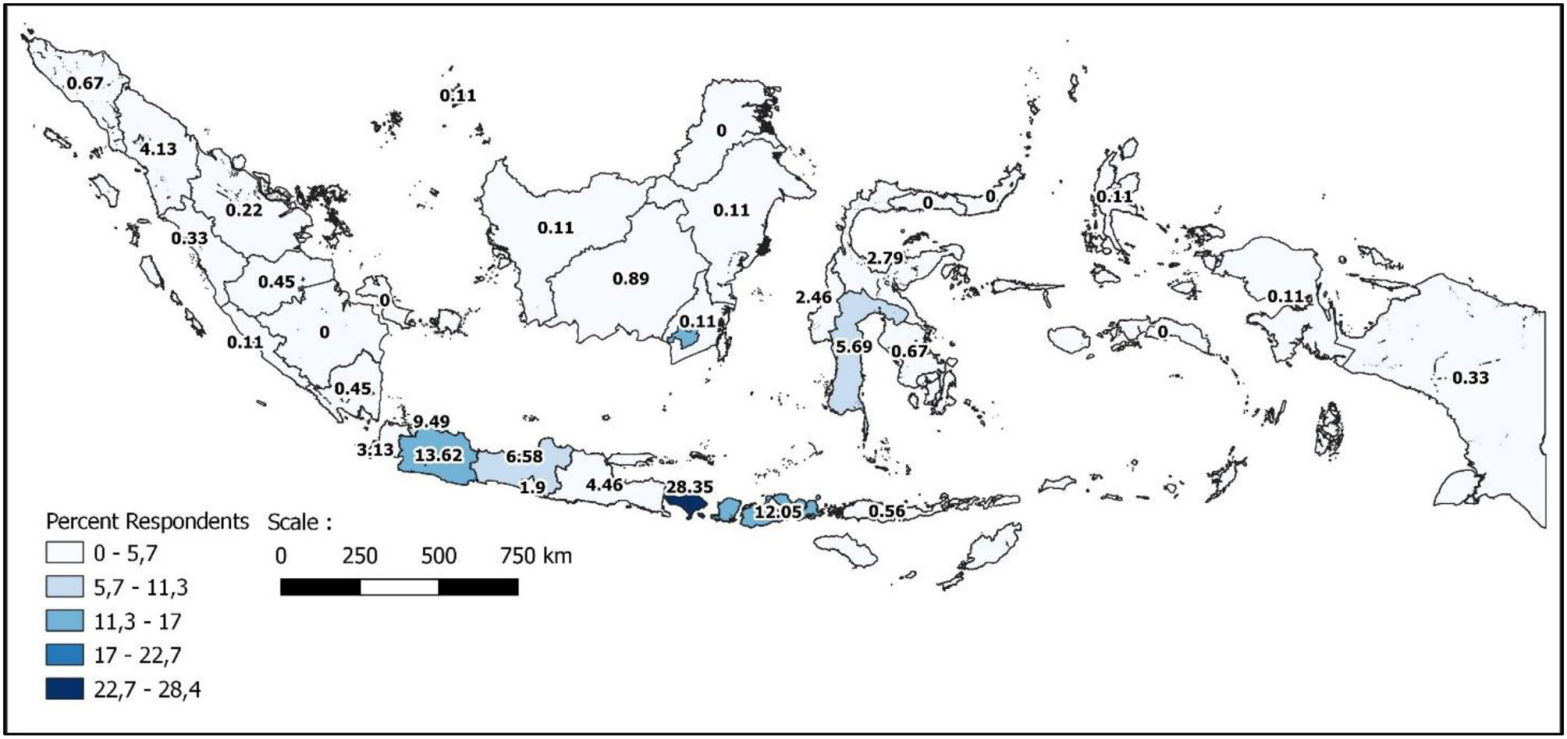
Geographical distribution of respondents.

**Figure 2:**
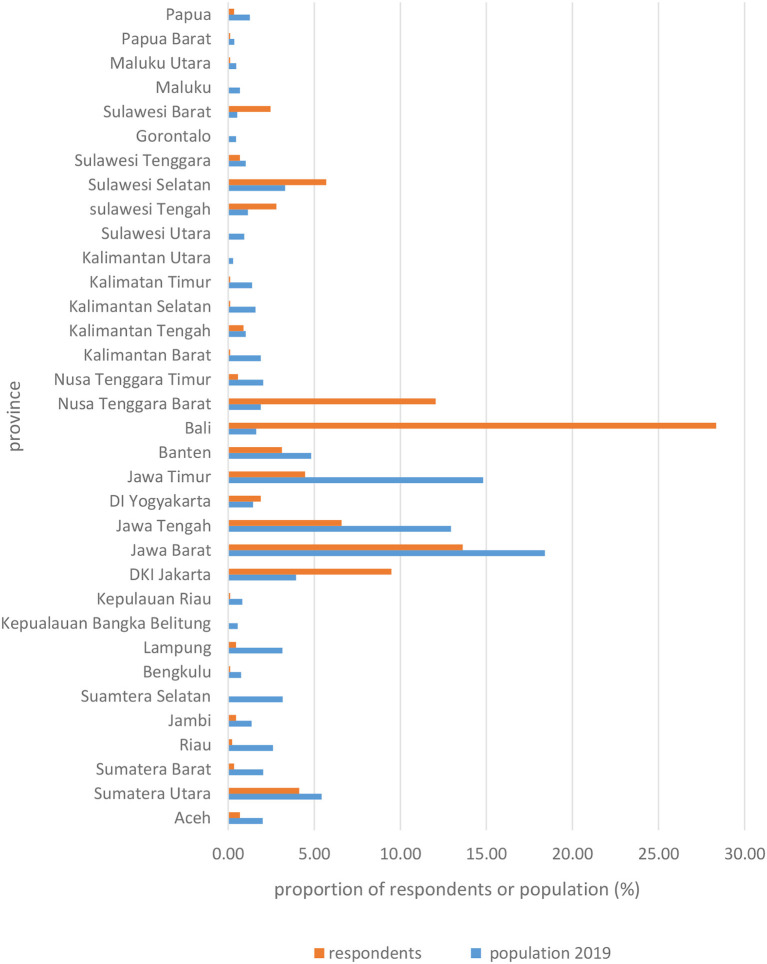
Comparison between respondents and population distribution (bottom).

The 896 respondents who participated in this survey represented many provinces of Indonesia and mostly originated from provinces with mid- level category of COVID-19 cases. The geographical distribution of respondents in comparison with the population distribution is presented in Figures 1, 2, showing that the respondent's distribution is not in line with the population distribution due to the nature of convenient sampling. The majority of respondents were from Bali (254 respondents) followed by West Java (122 respondents) and West Nusa Tenggara (108 respondents). Participant characteristics are presented in Table 1. Most of the respondents were from urban areas, were female (60%) and the mean age was 35 years old. The background education of the respondents ranged from primary to university level, with the majority of respondents having a university education background (75.11%). Family income ranged from under IDR 1 million (US$ 68.25) up to more than IDR 10 million (US$ 682,5) per month, with the majority of respondents earning from IDR 2.5 (US$ 170.61) up to IDR 5 million (US$ 341.23) per month (26.34%). Most of the respondents were employees in the private sector (36.61%) followed by students or unemployed persons (29.45%). The majority of respondents had a pipeline as their source of water (50.45 %), yet there was also a high percentage who sourced drinking water from a borehole (47.77%). While most of the respondents had never experienced water scarcity issues (67.08%), more than a quarter of respondents had experienced water scarcity for a few days, <10% of that over 2 days and <5% had experienced water scarcity over a couple of months in a year.

**Table 1:** Socio-demographic characteristic of respondents.

**Characteristics**	***n***	**%**
Settlement type		
Rural	246	27.46
Urban	650	72.54
Sex		
Female	543	60.60
Male	353	39.40
Age		
Mean (SD)	35.57	11.51
<20	82	9.15
21–30	266	29.44
31–40	210	23.44
41–50	235	26.23
>50	103	11.5
Education		
Primary	1	0.11
Secondary	222	24.77
University	673	75.11
Family income		
<IDR 1million US$ 68.25	144	16.07
IDR 1–2.5 million (US$ 68.25 - 170.61)	144	16.07
>IDR 2.5–5 million (US$ 170.61–341.23)	236	26.34
>IDR 5–10 million (US$ 341.23–682.5)	220	24.55
>IDR 10 million (US$ 682.5)	152	16.96
Occupation		
Entrepreneur	83	9.26
Employee	328	36.61
Teacher	15	1.67
Health workers	12	1.34
Government employee	195	21.76
Students/unemployed	263	29.35
Drinking water sources		
River, spring, rainwater	8	0.89
Drinking water vendor	8	0.89
Borehole	428	47.77
Pipeline	452	50.45
Water scarcity issue		
Never	601	67.08
A couple of days/year	196	21.88
A couple of weeks/year	59	6.58
A couple of months/year	40	4.46
Provincial-level of COVID-19 cases		
Low (<1%)	90	10.04
Medium (1-5%)	449	50.11
High (<5%)	357	39.84

### Handwashing Frequency

**Figure 3:**
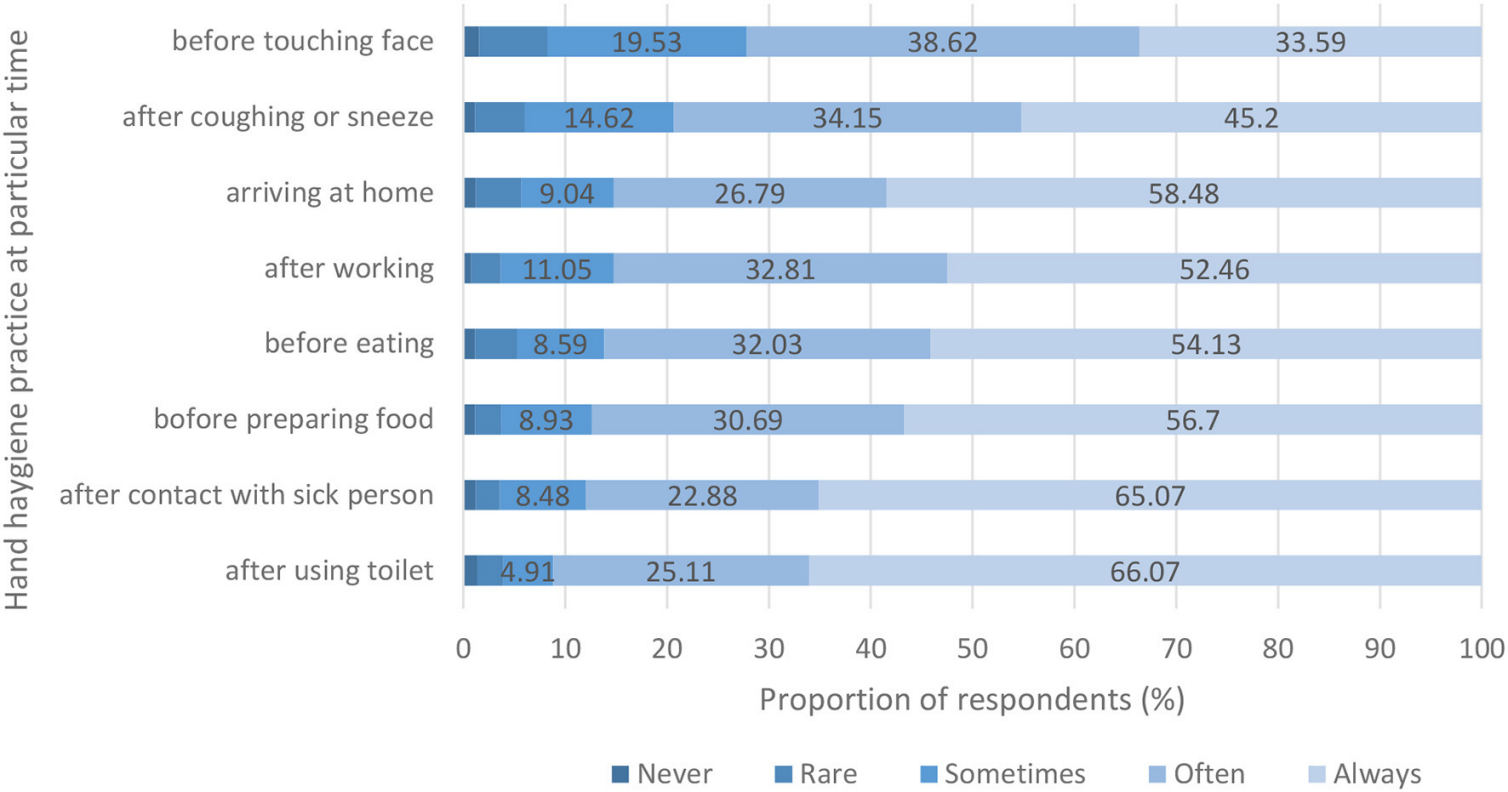
Respondents reporting hand hygiene practice at particular time with five different categories of frequencies.

**Figure 4:**
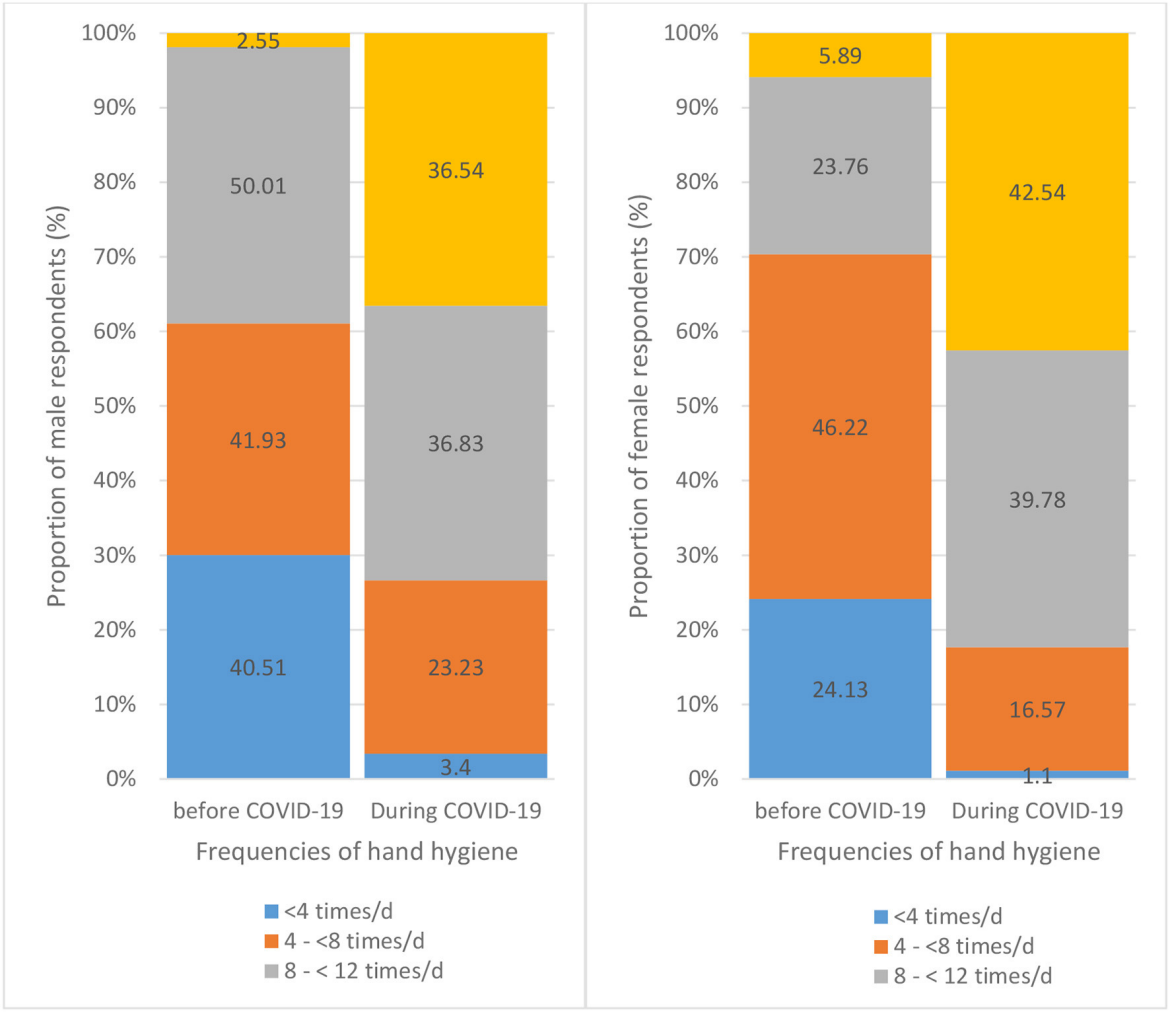
Change in hygiene frequencies from before to during COVID-19 pandemic for male group (left) and female group (right).

Female respondents reported higher handwashing frequencies than male respondents (p < 0.001) before and during the COVID-19 pandemic. There were 29.65 and 82.32% female respondents who reported handwashing frequencies of 8 times or more per day before and during the COVID-19 pandemic respectively, while there were 17.56 and 73.37% male respondents reporting handwashing practice with the same frequencies before and during the COVID-19 pandemic (Table 2). Female respondents also reported more handwashing frequencies before eating, when arriving home, after using the toilet, before preparing food, after working, after coming in contact with a sick person and after coughing or sneezing compared to male respondents during the COVID-19 pandemic (*p* < 0.05) (Supplementary Table 3). However, there was no significant difference between female and male respondents regarding handwashing practices before touching the face. Cleaning hands before touching the face and after coughing or sneezing were hand hygiene behaviors least frequently practiced (Figure 3). On the other hand, both female and male respondents (84.16 and 87.54% respectively) reported increased handwashing frequencies during the COVID-19 pandemic compared to pre-pandemic practices. Within each sex group, the increase in handwashing frequencies was statistically different between the time before and during the COVID-19 pandemic (*p* < 0.001) (Figure 4). There was also a difference in handwashing practices based on the education level of respondents. Respondents with a higher education level practiced handwashing more frequently than those with a lower education level (*p* < 0.05). Respondents with a higher education level also reported more frequent handwashing when arriving home (*p* < 0.005) and after using the toilet (*p* < 0.05) compared to those with a lower education level.

**Table 2:** Differences of hygiene behavior between male and female.

**Variables**	**Male**		**Female**		**Total**		***p*-value**
	***n***	**%**	***n***	**%**	***n***	**%**	
Hand hygiene frequencies before COVID-19							0.000
<4 times/d	143	40.51	131	24.13	274	30.58	
4– <8 times/d	148	41.93	251	46.22	399	44.53	
8– <12 times/d	53	15.01	129	23.76	182	20.31	
>12 times/d	9	2.55	32	5.89	41	4.58	
Hand hygiene frequencies during COVID-19							0.005
<4 times/d	12	3.4	6	1.1	18	2.01	
4– <8 times/d	82	23.23	90	16.57	172	19.2	
8– <12 times/d	130	36.83	216	39.78	346	38.62	
>12 times/d	129	36.54	231	42.54	360	40.18	
Reporting increase HH frequencies						0.161	
Yes	309	87.54	457	84.16	766	85.49	
No	44	12.46	86	15.84	130	14.51	
Cleaning surface before COVID-19							0.000
Never	20	5.67	11	2.03	31	3.46	
Rare	127	35.98	150	27.62	277	30.92	
Sometimes	144	40.79	245	45.12	389	43.42	
Often	51	14.45	120	22.1	171	19.08	
Always	11	3.12	17	3.13	28	19.08	
Cleaning surface during COVID-19							0.006
Never	5	1.42	0	0	5	0.56	
Rare	17	4.82	18	3.31	35	3.91	
Sometimes	80	22.66	96	17.68	176	19.64	
Often	196	55.52	321	59.12	517	57.7	
Always	55	5.58	108	19.89	163	18.19	
Intention to keep hygiene behavior post COVID							0.264
Low	20	5.7	22	4.1	42	4.7	
High	333	94.3	521	95.9	854	95.3	

*Total responses = 896; Significance of independence of samples indicated by Pearson Chi-Square test: p-value < 0.05*.

There were 95.4% of respondents who reported their intention to maintain their current handwashing frequencies when the COVID-19 pandemic ends, and there was no statistical difference between male and female respondents (Table 2). However, the intention was different between education level groups where respondents with high education level reported a stronger intention to maintain hand hygiene behavior after the COVID-19 pandemic ends compare to those with lower education level (*p* < 0.01). In regards to surface cleaning, there were 59.12 and 19.89% of female respondents who reported that they often and always practice surface cleaning at home during COVID-19, while 55.52 and 5.58% of male respondents reported the same practices (*p* < 0.05). These results indicate that sex and education level have modification effect on the handwashing frequencies of the respondents as well as intention to maintain the practice in post-pandemic era.

### Perceptions Related to Handwashing Behavior and Information Sources

In this study, 66.9% of respondents perceived that they have a medium to low risk of contracting COVID-19, and 65% of respondents perceived that they would only have mild to no symptom if they contracted COVID-19 (Table 3). On the other hand, many respondents perceived handwashing as an effective measure to prevent COVID-19 and other diseases (61.3%), and had less negative perceptions toward handwashing practice (77%). Regarding social norms, similar composition of respondents reported non-supportive perceived norms (52%) and supportive perceived norms (48%). When asked about barriers in accessing handwashing facilities, the majority (72.9%) reported less frequent barriers. Female and male respondents were only different regarding their perception toward susceptibility (*p* < 0.05) but were not different for other types of perceptions. In summary, even though many respondents perceived that they have low risk of COVID-19, they believe that hand washing is an effective measure for disease prevention and that their networks were supportive of the behavior.

**Table 3:** Respondents' perceptions toward hand washing behavior as preventive measure to contract COVID-19.

**Variables**	**Male**		**Female**		**Total**		***p*-value**
	***n***	**%**	***n***	**%**	***n***	**%**	
Perceived susceptibility							0.047
Very low risk	36	10.2	45	8.3	81	9.0	
Low risk	69	19.5	130	23.9	199	22.2	
Medium risk	117	33.1	202	37.2	319	35.6	
High risk	104	29.5	117	21.5	221	24.7	
Very high risk	27	7.6	49	9.0	76	8.5	
Perceived severity							0.660
No symptom	70	19.8	123	22.7	193	21.5	
Mild symptom	151	42.8	238	43.8	389	43.4	
Symptom that can affect daily activities	103	29.2	141	26.0	244	27.2	
Severe consequences	19	5.4	23	4.2	42	4.7	
Fatality	10	2.8	18	3.3	28	3.1	
Perceived effectiveness							0.306
Not effective	144	40.8	203	37.4	347	38.7	
Effective	209	59.2	340	62.6	549	61.3	
Negative attitude							0.644
More negative attitude	84	23.8	122	22.5	206	23.0	
Less negative attitude	269	76.2	421	77.5	690	77.0	
Perceived norm							0.250
Less positive norm	192	54.4	274	50.5	466	52.0	
More positive norm	161	45.6	269	49.5	430	48.0	
Perceived barriers							0.512
Frequent encounter barriers	100	28.3	143	26.3	243	27.1	
Rarely encounter barriers	253	71.7	400	73.7	653	72.9	

*Total responses = 896; Significance of independence of samples indicated by Pearson Chi-Square test : p-value < 0.05*.

### Factors Associated With the Frequency of Handwashing

In the multiple logistic regression, women were more likely to report a higher frequency of handwashing practice during the COVID-19 pandemic compared to men (OR 1.71, 95% CI 1.21–2.41) (Table 4). Respondents who perceived that they were susceptible to contracting COVID-19 disease were more like to wash their hand frequently (OR 7.08, 95% CI 2.26–22.17), as did respondents who perceived that handwashing is an effective measure to prevent diseases were more likely to practice frequent handwashing (OR 1.77, 95% CI 1.23–2.54). Moreover, respondents who had less negative attitude toward handwashing practice (handwashing is wasting water and time) reported more frequent handwashing compared to those with more negative perception (OR 1.93, 95% CI 1.32–2.82). Perceived norms were also found to be influential on handwashing frequency. Respondents who perceived more positive norms in their surrounding environment, where their friends and important people were also frequently practicing COVID-19 prevention behavior, where they felt it was necessary to provide good examples to others, and the perception that hand hygiene is a part of religious values, were more likely to frequently wash their hands (OR 1.68, 1.15–2.44). Interestingly, respondents who reported more experience with access or barriers to handwashing facilities were also more likely to wash their hand more frequently (OR 1.57, 95% CI 1.05–2.36) (Table 4). The findings show that in addition to sex, many psychosocial factors also have significant influence on the respondents' handwashing frequencies during the pandemic. On the other hand, education, age and perceived severity did not show significant association with the same practice.

**Table 4:** Factors influencing more frequent Hand Hygiene Practice (8 or more times per day) during COVID-19 pandemic.

**Variables**	**Univariate**			**Multivariate**		
	**OR**	**95% CI**		**OR**	**95% CI**	
Sex						
Male	1			1		
Female	1.69	1.22	2.33	1.71	1.21	2.41^*^
Age						
<20	1			1		
21–30	1.33	0.77	2.32	1.17	0.61	2.25
31–40	2.07	1.14	3.76	1.60	0.78	3.29
41–50	1.90	1.06	3.40	1.54	0.75	3.17
>50	1.23	0.64	2.35	1.08	0.49	2.41
Education						
Up to high school	1			1		
University	1.35	0.94	1.93	1.08	0.68	1.70
Perceived Susceptibility						
Very low risk	1			1		
Low risk	1.23	0.70	2.17	1.66	0.89	3.11
Mild risk	1.68	0.98	2.89	2.18	1.19	4.00^*^
High risk	2.02	1.13	3.62	2.21	1.15	4.25^*^
Very high risk	6.34	2.28	17.62	7.08	2.26	22.17^*^
Perceived severity						
No Symptom	1			1		
Mild symptom	0.76	0.50	1.17	0.78	0.49	1.25
Symptom that limit daily life	0.87	0.54	1.40	0.70	0.41	1.20
Severe symptom	0.98	0.42	2.28	0.70	0.29	1.74
Fatal (death)	1.06	0.38	2.96	0.38	0.12	1.25
Perceived effectiveness						
Not effective or not sure	1			1		
Effective	2.30	1.67	3.19	1.77	1.23	2.54^*^
Negative attitude						
More negative	1			1		
Less negative	2.37	1.67	3.36	1.93	1.32	2.82^*^
Perceived norm						
Less supportive norm	1			1		
More supportive norm	2.18	1.56	3.05	1.68	1.15	2.44^*^
Perceived barriers						
Frequent encounter barrier	1.30	0.90	1.90	1.57	1.05	2.36^*^
Rarely encounter barrier	1			1		

* *Statistics indicate significance of OR with p-value < 0.05. The p-value of Hosmer–Lameshow test was 0.705 and the classification table shows that model provide 79% correct prediction*.

## Discussion

This study examines the frequency of hand hygiene practices before and during the COVID-19 pandemic in Indonesia 10 weeks following the first announcement of social restrictions in Bali, and analyses the psychosocial factors affecting behaviors. The instrument used to measure hand hygiene behavior and relevant perceptions was deemed to have good validity and reliability according to Pearson validity test and Cronbach's Alpha reliability test. The results indicate that there is a significant increase in daily handwashing frequency during the COVID-19 pandemic reported by a majority of respondents. A study in United States (US) also found that respondents report handwashing practice more frequently than usual during the COVID-19 pandemic (27). Likewise, similar trends were found in daily handwashing frequencies during HINI influenza pandemic in Hong Kong, where 30.3% university students report increased hand hygiene frequency (23). Furthermore, the adoption of personal protective measures during the SARS pandemic in Hong Kong also increased considerably, however the practice decreased in the post-pandemic period (22). In our study, most respondents reported their intention to maintain their current hand hygiene practices when the pandemic ends. However, respondents with a lower education level reported less intention compared to those with higher education level. This indicates that a continuous promotion on hand hygiene after the pandemic is necessary to prevent future spread of diseases, targeting the population with lower education levels.

Consistent with previous studies, the results showed that there was a significant difference between sex in regards to daily hand hygiene practices during pandemic situations, where female respondents tend to report higher handwashing frequency per day compared to male respondents (23, 25, 27, 28). In our study, female respondents reported more hand hygiene practice at almost all critical points than male respondents except handwashing before touching the face. A previous study reported that university students were observed to touch their face 23 times per hour, suggesting a high frequency of face touching that involved contact with mucous membrane (such as the mouth, the nose and the eyes) that will increase the risk of infection, therefore hand hygiene compliance is a really important measure to prevent disease transmission (29). Our study also indicates that the difference in daily handwashing frequencies between male and female respondents is also consistent with findings before the pandemic situation. Thus, future hygiene promotion and disease prevention information should consider targeting males to improve the practice among this population group.

Regarding the perceptions relating to hand hygiene, this study shows that more than half of the respondents perceived that they are not susceptible to COVID-19 (no risk to medium risk) and perceived contracting only mild symptoms if infected. Similarly, a previous study during another pandemic also indicated that only a small percentage of respondents (7.7%) perceived a high or very high risk of being infected by the disease, and slightly fewer respondents (56.1%) compared to the proportion in our study perceived the possibility of having only mild symptoms from the disease (23). However, another study during early COVID-19 pandemic in US considered that respondents perceived that they have relatively high risk of being infected with a mean score of 43.6 ± 26.62 out of 100 (27). This perception also increased over time during the study period (27). Another study showed a higher risk perceived by Norwegian respondents where they were 60% likely being infected (28). These differences might results from varying health information and promotion exposures in the context of different countries, side from the effects from other demographic characteristic. Changes in perceptions can also occur over time depending on the fluctuation in pandemic situation in the country.

Many respondents in this study (61.3%) perceived that handwashing with soap is an effective prevention measure for COVID19 and other diseases. Previous studies reported that a higher percentage of respondents (93.3%) from the general population perceived frequent handwashing as an effective SARS prevention measure (25) and 95.7% of respondents from university students perceived that handwashing can prevent H1N1 influenza (23). The results of this study found that females are perceived themselves to be more susceptible of contracting the disease than male respondents, but both sexes did not show significant differences in regards to other perceptions. Park et al. (23) similarly found this difference in their study, but they also found that females and males had different perceptions toward severity if being infected and males are more likely than females to perceive handwashing with soap as effective. This difference could have resulted from differences in the respondents' characteristics and country context. As perception toward disease risk and effectiveness of behavior might vary over time and in different places during the pandemic, promotion messages need to be carefully designed to maintain the perception on the importance of hand hygiene in preventing diseases not only during, but also after the pandemic.

In this study, female respondents, respondents who perceived that they were more likely to be infected by COVID-19, respondents who perceived that handwashing with soap or with hand-sanitiser was an effective way to prevent COVID-19 transmission were more likely to frequently wash their hands during the COVID-19 pandemic. These findings are consistent with other studies stating that that sex (23), perceived susceptibility (24) and perceived effectiveness (23, 28) are predictors of preventive health behaviors. This study adds that the other types of perception related to negative attitudes toward behavior and perceived norm were significantly affecting handwashing frequency of the respondents during the pandemic situation. In this study, the perception that frequent handwashing can waste clean water and time, significantly predicts less frequent hygiene practice. Physical barriers such as water availability is one of the common barriers for handwashing practice, especially when water is limited (20) and thus may create this negative perception toward frequent handwashing practice. Social norms that are supportive toward the adoption of frequent handwashing practice and other preventive behavior were also found to influence practice, particularly the perception that their close friends and other important people were practicing the behavior more frequently during the COVID-19 pandemic. This has also been discussed in another study during the COVID-19 pandemic, that a group's behaviors and attitudes might explain increase in health protective behaviors (28). A previous study which was conducted in a non-pandemic situation indicated that social norms concerning the acceptance of hygiene water handling at home by all family members is a significant predictor to hygiene practice (30). Studies on the adoption of sanitation behaviors also highlight that social norms, such as where the perception that people in surrounding environments use toilets for defecating, can influence the adoption of similar behavior (31, 32). The social norms created after sanitation intervention related to unacceptance of open defecation has also improved community hygiene behavior (26). This finding highlights the importance to create a social norm, for example through community action as a potential promotion strategy to support the adoption of more hygienic practices in the community and in particular target groups.

Interestingly, perceived barriers in accessing handwashing facilities was more likely reported by respondents who reported more frequent handwashing practice. This could possibly be explained by the fact that respondents who frequently practice hand hygiene will be more concerned and more observant in searching for hand-washing facilities. Thus, they are more likely to notice this barrier compared to those who practice less frequent handwashing. Reducing this barrier and encouraging more people to wash their hands can be done through a small environmental modification known as nudging (21, 33). For example, since the COVID-19 pandemic, it was suggested to business and building managers to allocate hand-washing stations at the entrance of buildings in order to prompt good practice by visitors.

### Limitations of the Study

This study is limited in several ways. Firstly, a sampling bias may occur due to the way the survey was announced and distributed via social media. This distribution will highly depend on the social networks of the researcher which can caused an uneven geographical distribution of the respondents in this study. The survey also had limitations in that it was more likely to obtain responses from respondents who interested in the topic of hygiene, even though rewards were offered to reduce this selection bias. Moreover, respondents who did not have internet access and with primary education level or lower were uncaptured in the survey, thus the study result should be interpreted as limited to respondents without this characteristics. Compared to another study in Aceh, Indonesia, where the proportion of respondents with primary education level was 27% (34), while this study only captured 0.1% of this group. A follow-up study using a randomized recruitment design is planned to obtain a more representative sample.

Another limitation is related to the self-reporting nature of the survey, which might result in higher frequency of handwashing behavior reported by the respondents than the actual practice. For a comparison, a systematic literature review estimated that there were only 17% (95% CI 7–36%) of the population in South-East Asian countries who practiced handwashing with soap under non-pandemic conditions (35), while in this study, daily handwashing for eight or more times a day was reported by 17.56 % (male) and 29.65% (females) respondents before the pandemic. Measurement with Likert-scale was used in order to better capture variations in the behavioral outcomes and perceptions of respondents.

Despite the limitations, this study provides insights into the perception and hygiene behavior of the general population during the COVID-19 pandemic. In a pandemic situation, the communication of risk and promotion of preventive measures of COVID-19 transmission have been able to increase hygiene practices of the Indonesian population. This study also confirms the psychosocial factors that affect hand hygiene practices identified in other studies. Moreover, this study also adds that social norm is an important factor to encourage better compliance with handwashing practices. Thus, promotion strategies can be targeted to create this supportive norm to increase adoption and sustainability. Although understanding these psychosocial factors is important to design effective hygiene promotion strategies, other factors in the socio-ecological model of health are also crucial to be addressed to complement promotion strategies. As mentioned in the five action strategies of the Ottawa Charter, creating a supportive environment such as ensuring equity of water access and other supporting infrastructure as well as developing institutional and policy support to address social structural issues are necessary measures to more comprehensively address the issues of hygiene practice (36).

## Conclusion

In conclusion, the majority of respondents did increase their frequency of hand hygiene and reported handwashing of eight or more times each day during the ongoing COVID-19 pandemic. Sex, perceived susceptibility and effectiveness are important predictors of hand hygiene, which are similar to findings from previous studies in other pandemic contexts. This research highlights the importance of addressing the social norms that related to the perceived practice among friends and important people as a potential promotion strategy targeting specific groups by creating hand hygiene norms in the community. The findings also suggest the importance of eliminating barriers to access water and handwashing facilities to facilitate hygiene practices. Considering these factors that affect hygiene behavior is not only important to improve health promotion strategies during the pandemic, but also to improve promotion to sustain hand hygiene behavior after the pandemic as basic prevention measures, which is still crucial in developing countries.

## Data Availability Statement

The raw data supporting the conclusions of this article will be made available by the authors, without undue reservation.

## Ethics Statement

The studies involving human participants were reviewed and approved by Ethics Committee of Faculty of Medicine, Udayana University. The patients/participants provided their written informed consent to participate in this study.

## Author Contributions

ND and DL contributed to the study design and manuscript writing. ND and NH conducted the data analysis for the study. All authors contributed to the article and approved the submitted version.

## Conflict of Interest

The authors declare that the research was conducted in the absence of any commercial or financial relationships that could be construed as a potential conflict of interest.
