# Supplementary material for: Public Perception and Hand Hygiene Behavior During COVID-19 Pandemic in Indonesia
Source: Front Public Health. 2021 May 13;9:621800. doi: 10.3389/fpubh.2021.621800 (PMC8155304; doi:10.3389/fpubh.2021.621800)
Supplement: Supplementary file 1 [file Data_Sheet_1.docx]

**Supplementary material**

Supplementary Table 1 Summary of psychosocial factors, questions and possible answer assessed in the questionnaire

| Factor Groups |  | Questions | Answer |
| --- | --- | --- | --- |
| Behaviour | Hand washing frequencies during COVID-19 | The daily frequency with which I wash my hands with soap or use hand sanitizer during COVID-19 is… | <4 times, 4-<8times, 8-<12 times, >12 times |
|  | Hand washing frequencies before COVID-19 | The daily frequency with which I wash my hands with soap or use hand sanitizer before COVID-19 is… |  |
|  | Surface cleaning behaviour | The frequency with which I clean all surface at home during COVID-19, is… | Never, rare, sometimes, often, always |
|  | Intention post COVID-19 | I feel that it is necessary to maintain the same frequency and quality of handwashing after COVID-19 ends | Strongly disagree, disagree, neutral, agree, strongly agree |
| Risk Factors | Perceived susceptibility | Because of the environment where I live or my age or type of work, I feel that I am at risk of contracting COVID-19 | Very low risk, low risk, mild risk, high risk, very high risk |
|  | Perceived Severity | I feel that if I catch COVID-19 it is possible that I will experience symptoms of… | No symptom, mild symptom, symptom that limit daily life, severe symptom, fatal (death) |
| Attitude factors | Perceived effectiveness | I feel that handwashing with soap or using hand sanitizer are effective in preventing the spread of COVID-19 | Strongly disagree, disagree, neutral, agree, strongly agree |
|  |  | I feel that handwashing with soap or using hand sanitizer are effective in preventing the spread of other diseases such as diarrhea and cholera |  |
|  | Negative attitude | I feel that too often practicing handwashing is wasting water | Strongly disagree, disagree, neutral, agree, strongly agree |
|  |  | I feel that too often practicing handwashing is wasting time |  |
| Norm factors | Perceived norm | I feel that when the COVID-19 outbreak occurred: | Strongly disagree, disagree, neutral, agree, strongly agree |
|  |  | My friends or the closest people around me are more often wash their hands with soap |  |
|  |  | My friends or the closest people around me are maintaining social distance |  |
|  |  | My friends or the closest people around me are always wearing a face mask when going out |  |
|  |  | Community leader or important people to me (such as teacher, supervisor, religious leader, village leader, doctor) are more often wash their hands with soap |  |
|  |  | Community leader or important people to me (such as teacher, supervisor, religious leader, village leader, doctor) are maintaining social distance |  |
|  |  | Community leader or important people to me (such as teacher, supervisor, religious leader, village leader, doctor) are always wearing a face mask when going out |  |
|  |  | I have to provide a good example regarding handwashing with soap and maintain social distancing |  |
| Ability factor | Perceived barrier | When I am at home or going out during COVID-19, I have experienced that handwashing facilities were inadequate | Never, rare, sometimes, often, always |

Supplementary Table 2 Reliability and validity test results

| **Variables** | **Questions** | **Cronbach's alpha** | **Pearson correlation sig.** | **cut point based on median** |
| --- | --- | --- | --- | --- |
| perceived effectiveness | I feel that hand washing with soap or using hand sanitizer are effective in preventing the spread of COVID-19 | 0.856 | 0.000 | 7 |
|  | I feel that hand washing with soap or using hand sanitizer are effective in preventing the spread of other diseases such as diarrhea and cholera |  |  |  |
| perceived norm | I feel that when the COVID-19 outbreak occurred: | 0.854 |  | 27 |
|  | My friends or the closest people around me are more often wash their hands with soap |  | 0.000 |  |
|  | My friends or the closest people around me are maintaining social distance |  | 0.000 |  |
|  | My friends or the closest people around me are always wearing face mask when going out |  | 0.000 |  |
|  | Community leader or important people to me (such as teacher, supervisor, religious leader, village leader, doctor) are more often wash their hands with soap |  | 0.000 |  |
|  | Community leader or important people to me (such as teacher, supervisor, religious leader, village leader, doctor) are maintaining social distance |  | 0.000 |  |
|  | Community leader or important people to me (such as teacher, supervisor, religious leader, village leader, doctor) are always wearing face mask when going out |  | 0.000 |  |
|  | I have to provide a good example regarding hand washing with soap and maintain social distancing |  | 0.000 |  |
|  | I feel that hand washing with soap must be done because maintaining cleanliness is one of the religious value that need to be obeyed. |  | 0.000 |  |
| negative attitude | I feel that too often practicing hand washing is wasting water | 0.700 | 0.000 | 2 |
|  | I feel that too often practicing hand washing is wasting time |  | 0.000 |  |

Supplementary Table 3 Differences of hygiene behaviour during particular time between male and female

| **Variables** | **Male** |  | **Female** |  | **Total** |  | **p-value** |
| --- | --- | --- | --- | --- | --- | --- | --- |
|  | **n** | **%** | **n** | **%** | **n** | **%** |  |
| **Hand hygiene practice during particular time** | | |  |  |  |  |  |
| before eating |  |  |  |  |  |  | 0.000 |
| Never | 4 | 1.13 | 6 | 1.1 | 10 | 1.12 |  |
| Rare | 25 | 7.08 | 12 | 2.21 | 37 | 4.13 |  |
| Sometimes | 32 | 9.07 | 45 | 8.29 | 77 | 8.59 |  |
| Often | 134 | 37.96 | 153 | 28.18 | 287 | 32.03 |  |
| Always | 158 | 44.76 | 327 | 60.22 | 485 | 54.13 |  |
| arrived at home |  |  |  |  |  |  | 0.002 |
| Never | 4 | 1.13 | 7 | 1.29 | 11 | 1.23 |  |
| Rare | 21 | 5.95 | 19 | 3.5 | 40 | 4.46 |  |
| Sometimes | 40 | 11.33 | 41 | 7.55 | 81 | 9.04 |  |
| Often | 110 | 31.16 | 130 | 23.94 | 240 | 26.79 |  |
| Always | 178 | 50.42 | 346 | 63.72 | 524 | 58.48 |  |
| after using toilet |  |  |  |  |  |  | 0.000 |
| Never | 5 | 1.42 | 7 | 1.29 | 12 | 1.34 |  |
| Rare | 19 | 5.38 | 4 | 0.74 | 23 | 2.57 |  |
| Sometimes | 16 | 4.53 | 28 | May-16 | 44 | 4.91 |  |
| Often | 107 | 30.31 | 118 | 21.73 | 225 | 25.11 |  |
| Always | 206 | 58.36 | 386 | 71.09 | 592 | 66.07 |  |
| before preparing food |  |  |  |  |  |  | 0.000 |
| Never | 4 | 1.13 | 6 | 1.1 | 10 | 1.12 |  |
| Rare | 19 | 5.38 | 4 | 0.74 | 23 | 2.57 |  |
| Sometimes | 46 | 13.03 | 34 | 6.26 | 80 | 8.93 |  |
| Often | 127 | 35.98 | 148 | 27.26 | 275 | 30.69 |  |
| Always | 157 | 44.48 | 351 | 64.64 | 508 | 56.7 |  |
| after working |  |  |  |  |  |  | 0.009 |
| Never | 4 | 1.13 | 3 | 0.55 | 7 | 0.78 |  |
| Rare | 16 | 4.53 | 10 | 1.84 | 26 | 2.9 |  |
| Sometimes | 41 | 11.61 | 58 | 10.68 | 99 | 11.05 |  |
| Often | 129 | 36.54 | 165 | 30.39 | 294 | 32.81 |  |
| Always | 163 | 46.18 | 307 | 56.54 | 470 | 52.46 |  |
| after contact with sick person |  |  |  |  |  |  | 0.036 |
| Never | 7 | 1.98 | 4 | 0.74 | 11 | 1.23 |  |
| Rare | 13 | 3.68 | 8 | 1.47 | 21 | 2.34 |  |
| Sometimes | 30 | 8.5 | 46 | 8.47 | 76 | 8.48 |  |
| Often | 89 | 25.21 | 116 | 21.36 | 205 | 22.88 |  |
| Always | 214 | 60.62 | 369 | 67.96 | 583 | 65.07 |  |
| after coughing or sneezing |  |  |  |  |  |  | 0.035 |
| Never | 7 | 1.98 | 3 | 0.55 | 10 | 1.12 |  |
| Rare | 21 | 5.95 | 23 | 4.24 | 44 | 4.91 |  |
| Sometimes | 60 | 17 | 71 | 13.08 | 131 | 14.62 |  |
| Often | 122 | 34.56 | 184 | 33.89 | 306 | 34.15 |  |
| Always | 143 | 40.51 | 262 | 48.25 | 405 | 45.2 |  |
| before touching face |  |  |  |  |  |  | 0.426 |
| Never | 7 | 1.98 | 7 | 1.29 | 14 | 1.56 |  |
| Rare | 29 | 8.22 | 31 | 5.71 | 60 | 6.7 |  |
| Sometimes | 68 | 19.26 | 107 | 19.71 | 175 | 19.53 |  |
| Often | 139 | 39.38 | 207 | 38.12 | 346 | 38.62 |  |
| Always | 110 | 31.16 | 191 | 35.17 | 301 | 33.59 |  |
|  |  |  |  |  |  |  |  |
| Cleaning surface before COVID |  |  |  |  |  |  | 0.000 |
| Never | 20 | 5.67 | 11 | 2.03 | 31 | 3.46 |  |
| Rare | 127 | 35.98 | 150 | 27.62 | 277 | 30.92 |  |
| Sometimes | 144 | 40.79 | 245 | 45.12 | 389 | 43.42 |  |
| Often | 51 | 14.45 | 120 | 22.1 | 171 | 19.08 |  |
| Always | 11 | 3.12 | 17 | 3.13 | 28 | 19.08 |  |
|  |  |  |  |  |  |  |  |
| Cleaning surface during COVID |  |  |  |  |  |  | 0.006 |
| Never | 5 | 1.42 | 0 | 0 | 5 | 0.56 |  |
| Rare | 17 | 4.82 | 18 | 3.31 | 35 | 3.91 |  |
| Sometimes | 80 | 22.66 | 96 | 17.68 | 176 | 19.64 |  |
| Often | 196 | 55.52 | 321 | 59.12 | 517 | 57.7 |  |
| Always | 55 | 5.58 | 108 | 19.89 | 163 | 18.19 |  |
|  |  |  |  |  |  |  |  |
| Intention to keep hygiene behavior post COVID | | |  |  |  |  | 0.264 |
| Low | 20 | 5.7 | 22 | 4.1 | 42 | 4.7 |  |
| high | 333 | 94.3 | 521 | 95.9 | 854 | 95.3 |  |

Supplementary Table 4 Correlation between variables

|  | hand washing frequencies | Perceived Susceptibility | Perceived severity | Perceived effectiveness | Negative attitude | Perceived norm | Perceived barriers |
| --- | --- | --- | --- | --- | --- | --- | --- |
| hand washing frequencies | 1 | 0.136** | -0.004 | 0.170** | 0.164** | 0.154** | -0.046 |
| Perceived Susceptibility |  | 1 | 0.298** | 0.126** | 0.126** | 0.043 | -0.008 |
| Perceived severity |  |  | 1 | 0.031 | 0.026 | 0.019 | -0.022 |
| Perceived effectiveness |  |  |  | 1 | 0.126** | 0.337** | 0.046 |
| Negative attitude |  |  |  |  | 1 | 0.164** | 0.090** |
| Perceived norm |  |  |  |  |  | 1 | -0.002 |
| Perceived barriers |  |  |  |  |  |  | 1 |

**Spearman correlation coefficient significant at the 0.01 level (2-tailed)
